# Supplementary material for: Reduced Carbohydrate Diet Influence on Postprandial Glycemia—Results of a Short, CGM-Based, Interventional Study in Adolescents with Type 1 Diabetes
Source: Nutrients. 2022 Nov 5;14(21):4689. doi: 10.3390/nu14214689 (PMC9656657; doi:10.3390/nu14214689)
Supplement: Supplementary file 1 [file nutrients-14-04689-s001.zip › nutrients-1986744-supplementary.pdf]

Supplementary Materials:

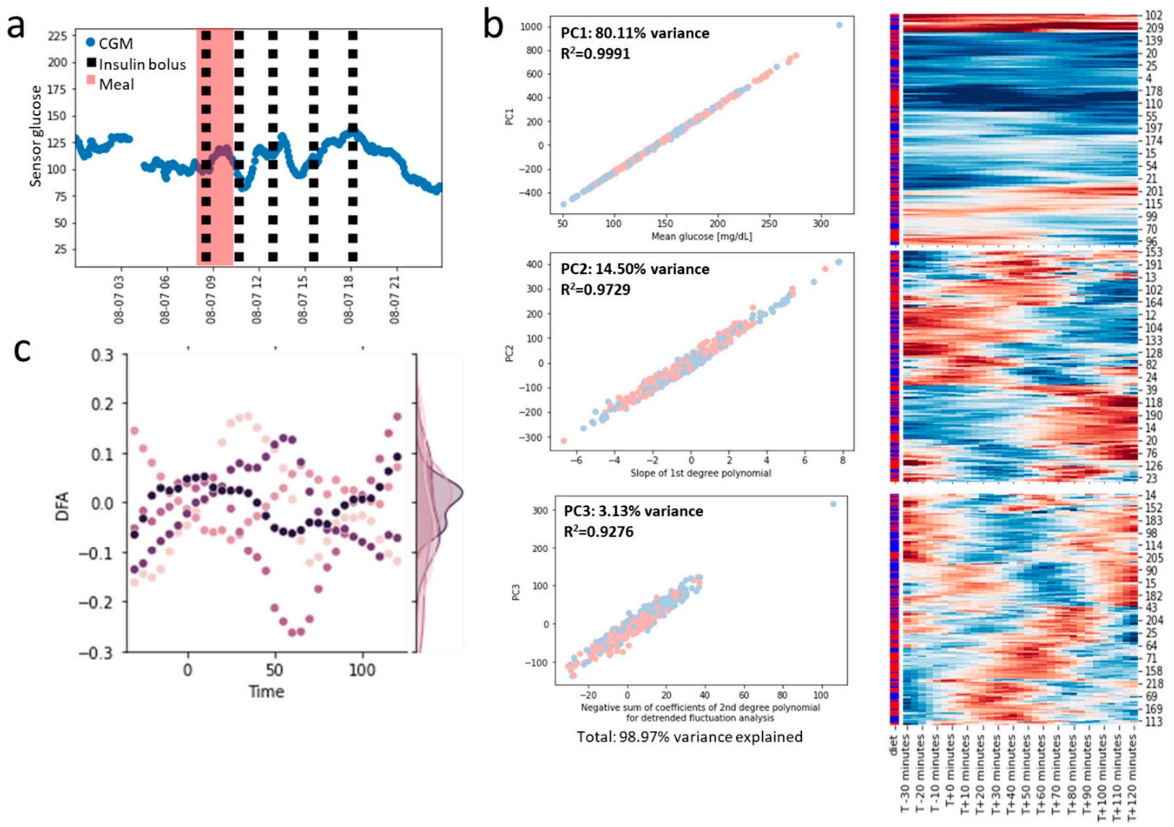

Figure S1. Analysis flowchart and reasoning. (a) Recovery of continuous glucose monitoring and insulin pump data for a meal in the trial cohort process was automated using approximated mealtime and paired CGM sensor-insulin pump data. Two independent researchers manually evaluated each record. 220 CGM-monitored meal responses were collected and successfully paired to meals. Next, principal component analysis was performed to determine sources of variability between collected records. (b) Weights from 1st, 2nd and 3rd principal components were mathematically evaluated and paired with most time-series functions: PC1 – mean of signal, PC2 – slope of 1st-degree polynomial, PC3 –coefficients of 2nd-degree polynomial. Heatmaps of raw sensor data (PC1), z-score for the record (PC2) and z-score after detrended fluctuation analysis (PC3) are provided for graphical representation of data. (c) 5 example records after detrended fluctuation analysis are plotted against time to demonstrate 2nd-degree polynomial-like behavior (PC3).

Table S1. Study group characteristics.

	All participants (n=26)
	**Median (25%-75%) or n (%)**
**Tanner score**	5 (4-5)
**Age [years]**	16.00 (14.00-17.00)
**TC [mg/dL]**	164.50 (140.00-180.00)
**Glucose disposal rate [mg/(kg × min)]**	6.08 (5.25-7.29)
**HDL [mg/dL]**	60.50 (50.00-70.00)
**LDL [mg/dL]**	85.00 (75.00-107.00)
**Vitamin D [ng/mL]**	21.65 (18.50-28.10)
**Initial HbA1c [mmol/mol]**	55.19 (50.82-59.56)
**Initial HbA1c [%]**	7.20 (6.80-7.60)
**Disease duration [years]**	6.00 (3.00-8.00)
**Mean daily insulin requirement [u/day/kg of weight]**	0.77 (0.59-0.96)
**BMI [centile]**	80.96 (57.95-89.94)
**TG [mg/L]**	70.50 (60.00-88.00)
**Time using pump [years]**	3.00 (2.00-7.00)
**Prehypertension**	6 (23.08)
**Hypertension**	3 (11.54)
**Body fat [%]**	21.85 (15.00-29.20)
**AST [IU/L]**	17.00 (15.00-19.00)
**ALAT [IU/L]**	13.50 (10.00-15.00)

Tables S2. Comparison of baseline continuous glucose monitoring (CGM) metrics between 30% and 50% carbohydrate diet.

CGM metric	30% carbohydrate diet Median (25%-75%)	50% carbohydrate diet Median (25%-75%)	p-value
**Time in target range (TIR) 70-180 mg/dL (3.9-10.0 mmol/l) (%)**	87 (73 – 94)	81 (77 – 89)	0.2804
**Time above target range(TAR) 180 - 250 mg/dL (10.0 - 13.9 mmol/L) (%)**	8 (3 – 20)	8 (4 – 16)	0.8093

Table S3. Pre- and postprandial glycemia for n=220 observations.

Meal	Diet	30 minutes prior, 120 minutes after meal		30 minutes prior meal		120 minutes after meal	
		Mean sensor glucose [mg/dL]	Coefficient of variation [%]	Mean sensor glucose [mg/dL]	Coefficient of variation [%]	Mean sensor glucose [mg/dL]	Coefficient of variation [%]
Breakfast (7:00 AM)	30% n=20	125.29± 35.79	15.02±6.67	122.75 ± 33.88	14.42±6.60	132.72 ± 50.77	2.55±1.87
	50% n=24	150.49±49.98	15.31±6.09	151.09±50.15	14.71±7.28	146.61±56.97	4.29±2.68
2nd Breakfast (10:00 AM)	30% n=21	136.86±26.10	11.61±6.44	137.27 ± 26.11	10.70±6.03	131.83±36.15	2.65±1.92
	50% n=27	116.03±40.74	18.61±8.43	115.83±40.16	17.09±7.66	110.70±54.33	4.98±3.55
Dinner (1:00 PM)	30% n=20	138.93±47.66	11.81±6.38	138.91±48.21	10.85±5.98	136.06±52.42	3.20±1.65
	50% n=24	118.14±42.48	16.14±8.49	118.61±42.76	15.78±8.58	113.44±47.11	3.64±3.30
Afternoon snack (4:00 PM)	30% n=20	144.63±47.19	9.00±4.44	144.38±46.86	8.60±4.29	144.40±52.54	1.98±1.45
	50% n=19	120.26±36.79	15.56±5.88	119.60±36.26	14.20±6.08	120.56±46.20	3.41±2.55
Supper (7:00 PM)	30% n=25	131.26±38.65	13.55±7.14	130.39±38.27	13.32±6.88	133.34±48.10	3.12±2.16
	50% n=20	135.00±40.67	13.96±7.81	136.46±43.85	12.31±8.16	124.29±38.36	3.75±3.08

Table S4. Meal and insulin-therapy related factors for meals in paired-meals analysis (n=128 records).

Meal	Diet	Caloric value [kcal] Mean ± SD	Meal weight [g] Mean ± SD	Hydrated carbon (CHO) [%g] Mean ± SD	Carbohydrate exchange unit Mean ± SD	Carbohydrates[g] Mean ± SD	Fat-protein exchange unit Mean ± SD	Fats[g] Mean ± SD	Proteins[g] Mean ± SD	Meal insulin requirements [U] Mean ± SD
**Breakfast (7:00 AM)**	**30% n=11**	413.64 ± 47.91	280.00 ± 51.19	35.45 ± 5.68	3.55 ± 0.57	35.45 ± 5.68	3.50 ± 0.33	19.09 ± 3.02	44.55 ± 1.51	4.72 ± 2.53
	**50% n=11**	415.45 ± 62.27	266.36 ± 58.53	49.09 ±9.44	5.05 ± 0.96	50.45 ± 9.61	2.27 ± 0.47	12.73 ± 4.67	19.09 ± 13.75	7.77 ± 3.73
**2nd Breakfast (10:00 AM)**	**30% n=15**	344.00 ± 141.90	182.00 ± 122.89	26.67 ± 12.49	2.77 ± 1.19	27.67 ± 11.93	1.97 ±1.44	5.67 ± 1.67	10.67 ± 1.76	3.69 ±3.32
	**50% n=15**	273.00 ± 39.77	200.67 ± 31.95	26.33 ± 7.19	2.47 ±0.67	24.67 ± 6.67	0.91 ± 0.10	5.00 ± 0.00	27.67 ± 2.58	3.63 ±1.66
**Dinner (1:00 PM)**	**30% n=10**	412.00 ± 166.39	297.50 ± 99.31	35.00 ±13.54	3.55 ±1.26	35.50 ± 12.57	3.06 ±1.39	27.00 ± 2.58	10.00 ± 0.00	4.39 ±2.01
	**50% n=13**	503.08 ± 56.33	442.31 ± 18.78	66.92 ±7.51	6.81 ±0.83	68.08 ± 8.30	2.85 ±0.09	20.00 ± 0.00	10.00 ± 0.00	8.37 ±4.38
**Afternoon snack (4:00 PM)**	**30% n=12**	195.83 ± 56.48	174.58 ± 30.86	20.83 ±5.15	2.13 ± 0.43	21.25 ± 4.33	1.09 ±0.18	5.42 ± 1.44	45.00 ± 0.00	2.62 ± 1.19
	**50% n=13**	172.31 ± 20.48	228.46 ± 19.08	23.08 ±4.08	2.15 ± 0.24	21.53 ± 2.40	0.88 ±0.10	5.00 ± 0.00	26.92 ± 2.53	2.62 ± 1.16
**Supper (7:00 PM)**	**30% n=13**	316.92 ± 46.79	156.15 ±35.95	29.62 ± 4.94	2.96 ±0.59	29.62 ± 5.94	1.17 ±0.58	8.46 ± 6.58	9.62 ± 1.39	3.28 ±2.02
	**50% n=15**	340.00 ± 37.80	210.67 ±18.31	45.33 ±9.15	4.23 ±0.53	42.33 ± 5.30	1.63 ±0.45	11.33 ±2.29	10.00 ± 0.00	4.09 ±2.66

Table S5. Number of meals from each patient taken for statistical analysis. Rows colored gray indicate paired meals.

	Breakfast n=22/44		2nd Breakfast n=30/48		Dinner n=23/44		Afternoon snack n=25/39		Supper n=28/45	
ID	30%	50%	30%	50%	30%	50%	30%	50%	30%	50%
1	1	1	0	2	0	2	1	1	1	0
2	0	0	0	0	0	0	3	0	0	0
3	2	0	2	1	0	1	2	0	1	0
4	3	1	2	0	1	1	0	0	1	0
5	0	1	2	1	1	0	0	2	2	2
6	0	0	1	0	0	0	1	2	1	2
7	1	1	1	1	2	0	0	0	2	0
8	0	1	0	2	1	3	1	1	0	1
9	1	0	1	0	2	0	1	2	0	3
10	2	2	1	0	1	0	1	1	1	0
11	1	2	1	2	1	1	1	1	2	1
12	0	1	0	0	0	1	1	0	1	1
13	2	0	1	1	0	1	1	1	1	1
14	1	0	2	2	0	3	0	1	1	0
15	0	1	0	3	1	1	0	0	1	3
16	2	0	0	2	0	1	0	2	2	1
17	2	1	0	1	0	2	2	0	1	2
19	0	0	1	2	2	1	1	1	0	0
21	0	2	0	0	1	1	0	0	1	0
22	1	3	1	2	0	0	1	1	0	1
23	0	2	0	1	1	2	0	0	0	0
24	1	0	0	0	2	0	0	0	1	0
25	0	2	1	2	1	1	1	1	2	0
27	0	1	0	1	1	2	0	1	2	2
29	0	0	1	0	0	0	0	0	0	0
30	0	2	3	1	2	0	2	1	1	0
